# Thermal, Rheological, and Surface Properties of Brewer’s Spent Grain and Its Oligo and Polysaccharides Fractions †

**DOI:** 10.3390/foods14244170

**Published:** 2025-12-05

**Authors:** Kalidas Mainali, Majher I. Sarker, Brajendra K. Sharma, Candice Ellison, Helen Ngo, Stefanie Simon, Madhav P. Yadav

**Affiliations:** Biobased and Other Animal Co-Products Research Unit, US Department of Agriculture, Agricultural Research Service, Eastern Regional Research Center, 600 E. Mermaid Lane, Wyndmoor, PA 19038, USAmajher.sarker@ysda.gov (M.I.S.); brajendra.sharma@usda.gov (B.K.S.);

**Keywords:** brewery spent grains, thermogravimetric analysis, rheology, zeta potential, scanning electron microscopy-energy dispersive X-ray spectroscopy

## Abstract

The brewing industry produces a large amount of byproducts, primarily brewery spent grain (BSG), which mainly consists of carbohydrates, proteins, and lipids. The different fractions isolated and extracted from BSG have significant potential for waste valorization and could be used as functional products or food ingredients. In this study, specific BSG-derived fractions (Hemicellulose A, Hemicellulose B, and oligosaccharides) were isolated and characterized to evaluate their potential applications. Thermogravimetric analysis data showed that the residue at 600 °C for various fractions is approximately 20% under N_2_, compared to 10% in air for BSG fractions. The rheological properties of Hemicellulose A and Hemicellulose B fractions from brewers’ spent grain (BSG) exhibit high viscosity, suggesting a strong dependence on molecular weight. This characteristic implies that their elevated molecular size may play a key role in their capacity to form gels, potentially enhancing their functionality in applications requiring thickening or structural integrity. Among the BSG fractions, Hemi. It had a viscosity of >102 mPa s^−1^ at a 3% (*w*/*v*) concentration, which was higher than Hemi B and oligosaccharides at the same concentration. The zeta potential of BSG fractions at various pH and concentrations was measured to assess the effects of pH and concentration. Additionally, scanning electron microscopy-energy dispersive X-ray spectroscopy (SEM-EDX) revealed the surface morphology and composition of each fraction. The highest Phosphorus (P) (%) was found on the surfaces of both Hemi B and the hexane-extracted BSG. The surface elements of each fraction primarily included C, O, N, P, Ca, and Mg.

## 1. Introduction

In 2021, global beer production reached 186 gigalitres. For every 100 L of beer brewed, approximately 20 kg of wet brewer’s spent grain (BSG) is generated [1,2,3]. This byproduct accounts for about 85% of the total waste produced during beer manufacturing, making it the most significant waste stream by mass. While this ratio can vary slightly depending on brewing style, wort gravity, and technological processes, BSG remains the dominant residue across most brewing operations [3,4,5]. Alcoholic beverage production contributes significantly to the economy of many countries [4,5,6]. Beer is a fermented beverage made from malted grains, water, yeast, and hops, and is among the most widely consumed beverages worldwide [1,7,8]. During preparation, barley grains undergo multiple steps before the initial brewing stage, which involves mashing and malting, and fermentation to ethanol. The latter procedure promotes the malt constituents’ enzymatic hydrolysis until their breakdown products are generated [7,9]. The goal of mashing barley starch is a process that breaks carbohydrate molecular chains into simple sugars that can be converted via enzymatic degradation [10,11]. The procedure produces a sweet fluid that is drained through the malted barley grain, which has not been broken down, and is then utilized as the fermentation medium to make beer. Brewers’ spent grains (BSG) are the solid portion that is left behind after removing the wort. BSG is the most abundant side-stream solid product of the brewing industry. It has 80% moisture and large amounts of fiber, including cellulose, hemicellulose, lignin, protein, starch, and lipid [10,12,13,14].

The brewing business plays a significant role in the global economy, but it also poses environmental challenges due to the substantial generation of BSG waste. The protein content of BSG typically ranges between 18 and 30 (wt.%), and its concentration is influenced by several key factors, such as selection of raw grains, the use of specific brewing additives, and the operational parameters employed during the brewing process [13,15]. Cellulose (glucose polymer) and hemicellulose (arabinoxylan) are the two main polysaccharide constituents in BSG [16,17,18]. However, the high moisture content and complex composition make it highly susceptible to microbial growth, making it challenging to store and transport

Due to its complex composition and high annual output, BSG is a low-cost, abundantly available material, which has generated significant interest for further research into its potential valorization [19,20]. Rich in cellulose and non-cellulosic polysaccharides, BSG can be broken down into useful components through various methods, such as diluted acid, alkali, supercritical CO_2_, and steam explosion. Although several disposal methods, like composting, landfilling, animal feeds, and anaerobic digestion, have been reported in the literature [21,22,23], each method has drawbacks, such as extensive land requirements, long reaction times, low efficiency, and CO_2_ emissions. Moreover, these methods are not sustainable because they release toxic gases and lead to water and soil pollution [24,25].

Thermochemical processes like pyrolysis offer promising alternatives. Thermochemical conversion methods, like pyrolysis and hydrothermal liquefaction/carbonization, have the potential to convert BSG into various products. Pyrolysis yields three main products: liquid organics (such as bio-oil, which includes a complex mix of oxygenated organics), solid residues (biochar), and non-condensable gases (like CO, H_2_, CH_4_, and light hydrocarbons). These products are suitable for various applications [26,27]. Additionally, hydrothermal carbonization is a thermal valorization process that uses wet BSG directly at elevated temperatures (typically 200 to 260 °C) in subcritical water and produces solid fuel [27,28].

Proper management of BSG is crucial for breweries, and finding effective, environmentally friendly ways to use or dispose of this by-product can help reduce waste and the environmental impact of the brewing industry. Alternatively, separating BSG into its constituent components followed by their detailed characterization will help in exploring their potential applications in food and non-food industries. Using agricultural and food waste to produce highly functional materials with desirable properties can address both waste management and transportation issues [1,2,3].

In our previous study [13], we reported the separation, yield, and physicochemical properties of four main fractions of BSG. The objective of the current research is to conduct a detailed characterization of these fractions and study their thermal behaviors, surface properties, and rheological properties. Furthermore, we have also measured the surface charge of particles under different conditions, which evaluates the long-term stability of these fractions under different pH conditions. These studies may contribute towards the identification of these fractions’ applications as a thickener, gel former, or flavor emulsifier in soft drinks and many food products, which will be desirable and appealing to consumers.

## 2. Materials and Methods

### 2.1. Materials

Brewery spent grain (BSG) was obtained from the “Iron Hill Brewery” located in Exton, PA. The collected BSG was oven-dried at 105 °C for 24 h in a vacuum oven. The dried sample was then ground into small particles (<0.5 mm). The ground BSG was de-oiled using hexane, as described in the literature [29]. In brief, about 300 g of ground BSG was added to 2 L of hexane at room temperature and stirred mechanically for 1 h. The suspension was vacuum filtered to remove the hexane, and the BSG residue was dried in a vacuum oven at 55 °C for 2 days, which was fractionated in an alkaline solution, as previously reported [13,29]. Alpha amylase used in this process was sourced from Novozymes North America Inc., located at 77 Perry Chapel Church Rd., Box 576, Franklinton, NC 27525. The details of this study can be found in our previous publication [13].

### 2.2. Thermal Behavior of BSG and Its Fractions

The thermal behavior of BSG and its fractions was examined under nitrogen and air atmospheres. For thermogravimetric analysis (TA Q500 V20.13 Build 39, New Castle, DE, USA) thermal analyzer was used. Approximately 5–10 mg of each sample was heated under N_2_ atmosphere (balance gas: 40 mL/min; sample gas: 60 mL/min) from room temperature to 600 °C at a rate of 20 °C/min. The TGA tests were repeated under the same conditions in an air environment (60 mL/min). All tests were performed at least three times. Weight loss and temperature shifts during the thermogravimetric (TGA) and differential thermogravimetric (DTG) analyses were recorded. To reduce variations in heat and mass transfer, the weights of BSG and its fractions were kept around 10 mg.

### 2.3. Scanning Electron Microscopy with Energy-Dispersive X-Ray Spectroscopy (SEM-EDX) Analysis

The topography and microstructure of BSG and its fractions were analyzed by SEM-EDX. The sample particles were adhered to carbon tape on aluminum stubs, gold-coated with an EMS 150R ES Sputter Coater (Electron Microscopy Sciences, Hatfield, PA, USA) for 60 min and viewed in a field electron ion company (FEI) with field emission gun (FEG), 200F scanning electron microscope at 10 kV, spot 3. Furthermore, Aztec software and an Oxford X-Max80 detector (Oxford Instruments, High Wycombe, UK) were used for elemental mapping. The elemental mapping data were acquired at 20 kV spot 5.

### 2.4. Rheological Measurement

For studying rheological and Zeta potential properties, 1, 2, and 3% solutions of Hemi A, Hemi B, and Oligosaccharides in water were prepared by stirring them at room temperature for 12 h. The solutions of each sample at each concentration were prepared at pH 5, 7, and 10. The viscosity and rheological properties of each sample solution were measured using an Anton Parr Modular Compact Rheometer (MCR 102) equipped with a circulating bath, an electronically controlled heating system, and the concentric cylinder geometry [30]. The steady-state viscosity of all samples was measured at 25 °C by varying the shear rate from 1 to 100 s^−1^.

### 2.5. Zeta Potential (§)

The zeta potential of the solution of each BSG fraction was measured using a Nano-Zetasizer 3000 (Malvern Instruments Ltd., Malvern, UK) as reported in the literature [31]. All the measurements were performed by maintaining constant tension (100 V) as recommended for a suspension having an EC lower than 1 mScm^−1^. Three independent zeta potential measurements were conducted for each sample to ensure reproducibility, and the mean value was reported as representative of the sample’s electrokinetic profile.

### 2.6. Statistical Analysis

All measurements were made at least three times. The standard deviation and mean were computed in an Excel spreadsheet. The values in parentheses indicate the standard error of the mean value.

## 3. Results and Discussions

**Figure 1:**
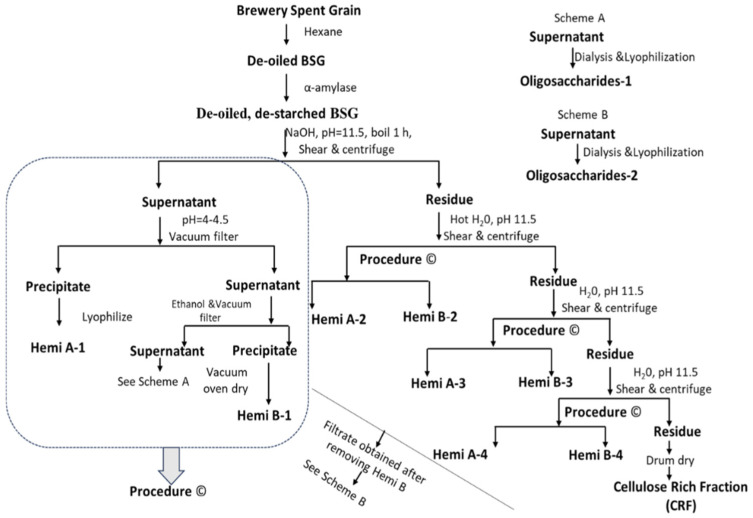
General scheme for brewery spent grains (BSG) fractionation and isolation [13].

Hemicellulose A, Hemicellulose B, cellulose-rich fraction (CRF), and Oligosaccharides were extracted from the hexane-extracted BSG as shown in the scheme below (Figure 1) following our earlier work [13], which gives the details of the separation process and the yield of each fraction, including the oligosaccharides before and after dialysis. As reported in the scheme, the main fraction of Hemi. A and Hemi. B was isolated from the de-oiled and de-starched BSG supernatant. The final residue remaining after the extraction of Hemicellulose A and B was suspended in water and drum dried to produce the cellulose-rich fraction (CRF). For each hemicellulose fraction, the supernatant was evaporated nearly to dryness, redissolved in water, and dialyzed against deionized water using 1 kDa molecular weight cut-off tubing for four days, with the water replaced three times daily. Following dialysis, the solution was concentrated and lyophilized to yield a purified, soft material referred to as oligosaccharides. Due to the high salt content in the oligosaccharide fraction, dialysis was performed using a 1 kDa molecular weight cut-off membrane, effectively removing the majority of the salt [13].

### 3.1. Thermogravimetric (TGA) and Derivative Thermogravimetric (DTG) Analysis

**Figure 2:**
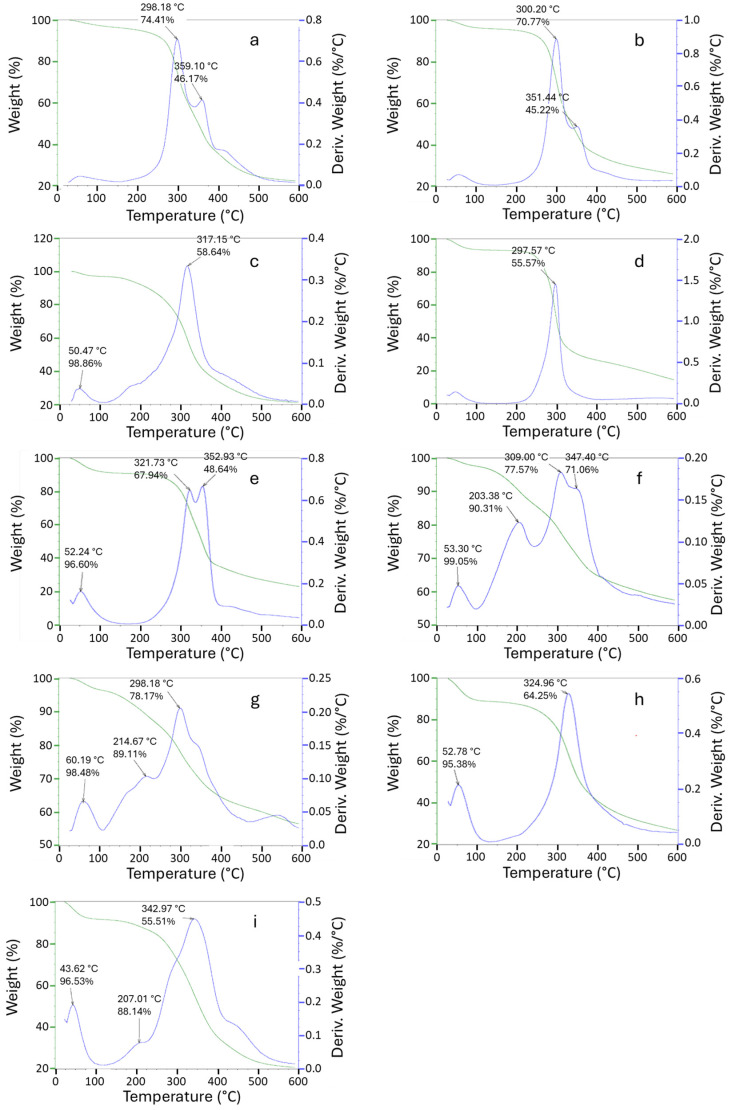
TGA/DTG of BSG and its fractions in the presence of N_2_: (**a**) Original BSG; (**b**) Hexane extracted BSG; (**c**) Hemi-A; (**d**) Hemi-B; (**e**) CRF; (**f**) Oligo-1; (**g**) Oligo-2; (**h**) Oligo-1- after dialysis; (**i**) Oligo-2- after dialysis.

The TGA data for studying the thermal behavior of BSG and its fractions were obtained at a heating rate of 20 °C/min. Figure 2 shows the thermogravimetric analysis (TGA) and derivative thermogravimetric (DTG) profiles of all samples. Each fraction displays a distinct thermal decomposition stage, including dehydration, decomposition, and devolatilization. TGA curves provide a mass loss ratio, which is related to the fraction of essential constituents. As reported in the literature [9,13,32], the mass loss peak around 300 °C in both original BSG and hexane extracted BSG, corresponding to the hemicellulose decomposition temperature (original BSG at 298.2 °C and hexane extracted BSG at 300.2 °C), with residue masses of 74.4% and 70.8%, respectively (Figure 2a,b). While there is some moderate reduction in residue masses in the hexane-extracted BSG, the main difference between the hexane-extracted BSG residue and the original BSG is the reduction in the high-temperature peak around 415 °C, which suggests that hexane extraction was important for removing its oily substances, which decompose at high temperatures.

The Hemi. A and Hemi. B fractions show their first major decomposition occurring at 317.2 °C and 296 °C, with residue masses of 58.6% and 55.6%, respectively (Figure 2c,d). However, Hemi B exhibits a sharp decomposition peak while Hemi A shows a broader thermal event, suggesting that Hemi B may be of greater purity, as sharper peaks are indicative of more homogeneous materials. Although Hemi A exhibits a higher peak decomposition temperature, its thermal degradation begins around 150 °C, significantly earlier than Hemi B, which starts around 230 °C. This earlier onset indicates that Hemi B may be more thermally stable overall. In the case of CRF, cellulose decomposition began at 220 °C and peaked at 321.7 °C, with a residue mass of 67.9% (Figure 2e). The early decomposition of CRF may be associated with the intrinsic moisture content in cellulose.

The thermal behavior of oligosaccharides appears to be slightly different, exhibiting less overall mass loss by the end of the thermal cycle compared to the other fractions (Figure 2f,g). While the other fractions decomposed to 20–30% of their original mass, the oligosaccharide fractions retained 55–60% of their initial mass. Further, the oligosaccharides exhibited an earlier decomposition onset than the other fractions. Oligo-1 started to decompose at approximately 130 °C with its first peak at 203.4 °C, corresponding to a residue mass of 90.3%. The onset of decomposition for Oligo-2 was similar to Oligo-1, but its peak was slightly later at 214.7 °C, corresponding to a residue mass of 89.1%. The early decomposition at lower temperatures corresponds to low-molecular-weight structures with loosely bound C-C bonds. The extraction processes for Oligo 1 and Oligo 2 were slightly different, which may account for the slightly different thermal behavior. As shown in Figure 1, Oligo 1 was recovered from the supernatant obtained from the Hemi. B-1 precipitate collection, while Oligo 2 was recovered from the supernatant of Hemi. B-2 precipitate collection [13]. DTG analysis of Oligo 1 and 2 was conducted before and after dialysis for comparison. The DTG curves clearly show that dialysis removed most of the salt and impurities from the Oligo 1 sample (Figure 2h), resulting in a more uniform curve. A similar pattern in the DTG analysis curves was observed for Oligo-2 (Figure 2i), both before and after dialysis.

**Figure 3:**
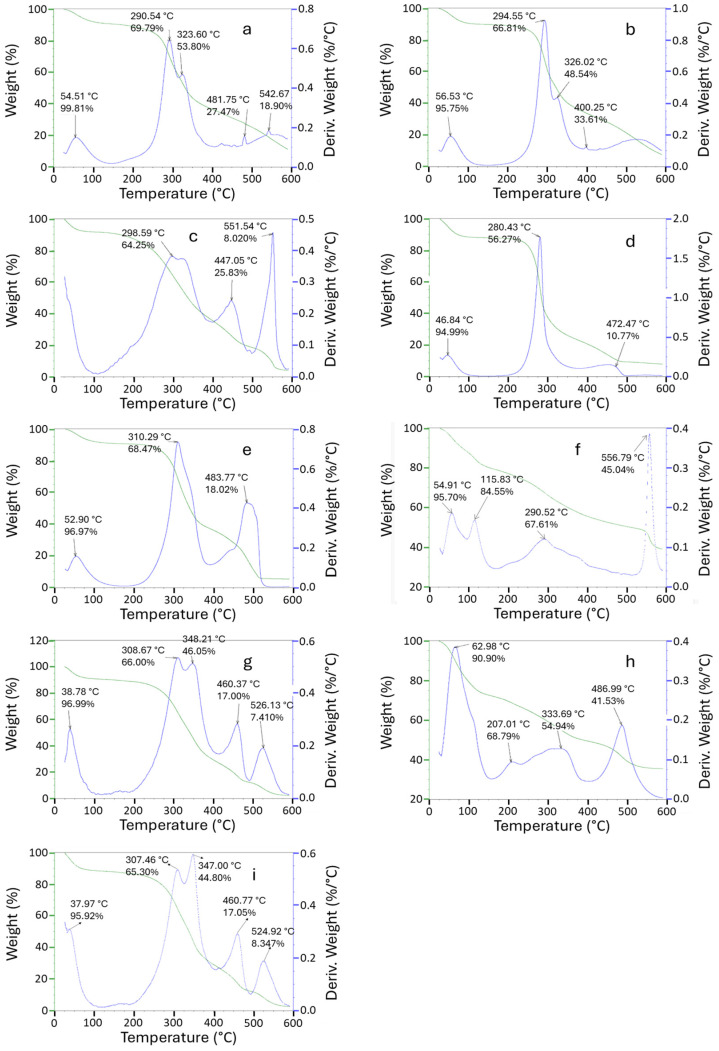
TGA/DTG of BSG and its fractions in the presence of air: (**a**) BSG; (**b**) Hexane extracted BSG; (**c**) Hemi-A; (**d**) Hemi-B; (**e**) CRF; (**f**) Oligo-1; (**g**) Oligo-2; (**h**) Oligo-1 after dialysis; and (**i**) Oligo-2 after dialysis.

In the presence of air, the thermal behavior of BSG and its fractions exhibits three distinct stages. In the first stage, the BSG displays dehydration at 54.5 °C, with a residue mass of approximately 96.8% (Figure 3a), indicating that the original hexane-un-treated BSG has 3.2 wt.% moisture. The second stage is related to oxidative degradation, and the final stage, which occurs between 300 and 600 °C, results in complete devolatilization. Moreover, the hexane-extracted BSG (Figure 3b) exhibited a DTG profile such as the original BSG, with a slightly higher oxidative degradation temperature, corresponding to hemicellulose (294.6 °C) and cellulose (326 °C). Again, the variation in the oxidative degradation temperature of these two samples is likely due to the removal of oily substances from the original BSG by hexane extraction.

The DTG curves of both Hemi. A and Hemi. B (Figure 3c,d), show that their maximum oxidative decomposition occurs at 298.6 °C and 280.4 °C, respectively. The DTG profiles of both Hemi A and Hemi B look different in an air environment. This observation concludes that Hemi. A has a loosely bound chemical structure (referring to multiple peaks), while Hemi B is more likely to be a compact single component, called arabinoxylan. A similar behavior was observed for Hemi B under inert conditions (Figure 2d), consistent with the superior purity of the Hemi B fraction compared to Hemi A. The multiple peaks in Hemi. A’s DTG indicates that it is more prone to oxidative degradation compared to the single arabinoxylan polymer present in Hemi. B. Similarly, the CRF exhibits a single oxidative degradation peak showing one main component, which is cellulose (Figure 3e). Under oxidative conditions in air, all peak temperatures shift to lower values, reflecting the endothermic nature of oxidative thermal degradation and the greater susceptibility to early degradation. These signature peaks showed that the BSG fractions are prone to thermal degradation in an air environment. Shapiro et al. (2023) [33] demonstrated that a better separation of peaks is responsible for different components in the presence of air, and we are also observing multiple peaks in an oxidative environment (i.e., under air).

Furthermore, the DTG curves of oligosaccharides show decomposition at a lower temperature (early decomposition), indicating their low molecular weight. These low molecular weight oligosaccharides have the potential to be used as prebiotics for promoting gut health. The multiple residual DTG peaks in each fraction showed a polymeric complex structure of the respective fractions. It was found that the residue at 600 °C for various fractions is higher (15–20%) under nitrogen compared to air (5–10%). This 5–10% residue under air is mostly due to ash or maybe some hard coke, while 15–20% residue under nitrogen may reflect the potential of soft coke/biochar (including ash) from these fractions [7,23,33]. Overall, it is beneficial to understand the thermal stability of these BSG fractions to explore their application in different food products.

### 3.2. SEM-EDX Analysis

**Figure 4:**
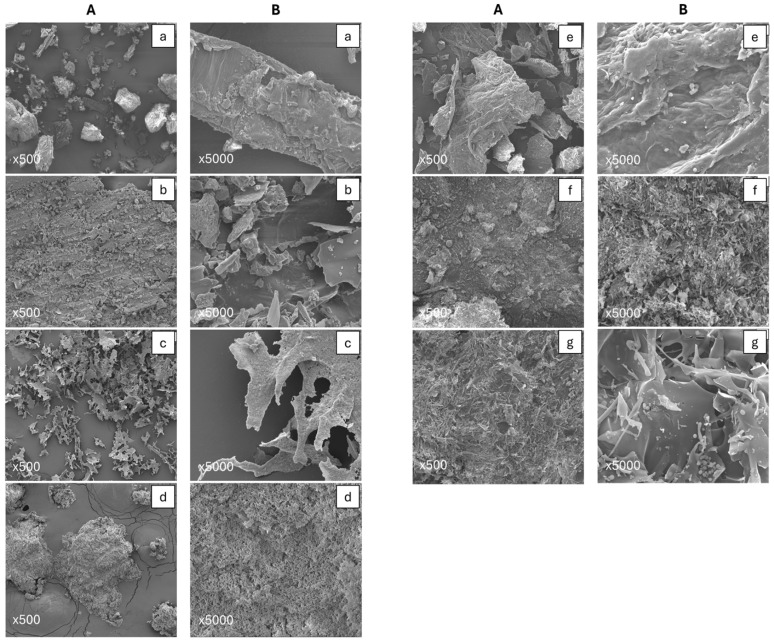
SEM images of BSG and its fractions, (**A**): 500× and (**B**): 5000×. (**a**) BSG; (**b**) Hexane extracted BSG; (**c**) Hemi A; (**d**) Hemi B; (**e**) CRF; (**f**) Oligo-1 after dialysis; and (**g**) Oligo-2 after dialysis.

SEM-EDX analysis was conducted to examine the surface morphology of BSG and its different isolated fractions. The SEM images of each BSG fraction display the formation of spherical clusters spread across the surface (Figure 4). In all BSG fractions, a higher magnification image (40,000×) confirms the presence of microporous cavities (see Figure S1). In most cases, these fractions show a porous, rough, and fluffy structure. These features suggest that these BSG fractions could be used as porous materials or could be modified to develop such materials, which have a wide range of applications, such as adsorbents and catalysis, among others [3,27].

The surface composition analysis of BSG and its fraction was performed using SEM-EDX. It shows that C, O, Ca, and Mg are their primary elemental constituents (Table 1). The details of SEM-EDX elemental composition spectra are given as Supplementary Information (Figure S2). As compared to our previous research (bulk properties), the surface composition trends follow a slightly uniform composition in each fraction [13]. The high carbon content was detected in Hemi A and CRF. These carbon-rich fractions can be used in the thermochemical conversion processes to produce carbon-rich biochar and bio-oils [27]. The high N content in both Oligo 1 and 2, Hemi A, and hexane-extracted BSG fraction indicates that these fractions can be used as N precursors/additives. Similar results were reported for these fractions earlier using elemental analysis data [13]. Fractions with high N content can be used as functional materials, biosorbents for various applications [3], while the fractions with rich minerals, such as phosphorus, calcium, manganese, and silicon, could serve as nutrients for yeasts, which promote bioconversion processes [34,35]. Overall, this study demonstrates that each of the BSG fractions not only has a unique chemical composition but also has different surface and bulk properties, as shown in SEM micrographs.

### 3.3. Shear Rate Dependence of Viscosity

**Figure 5:**
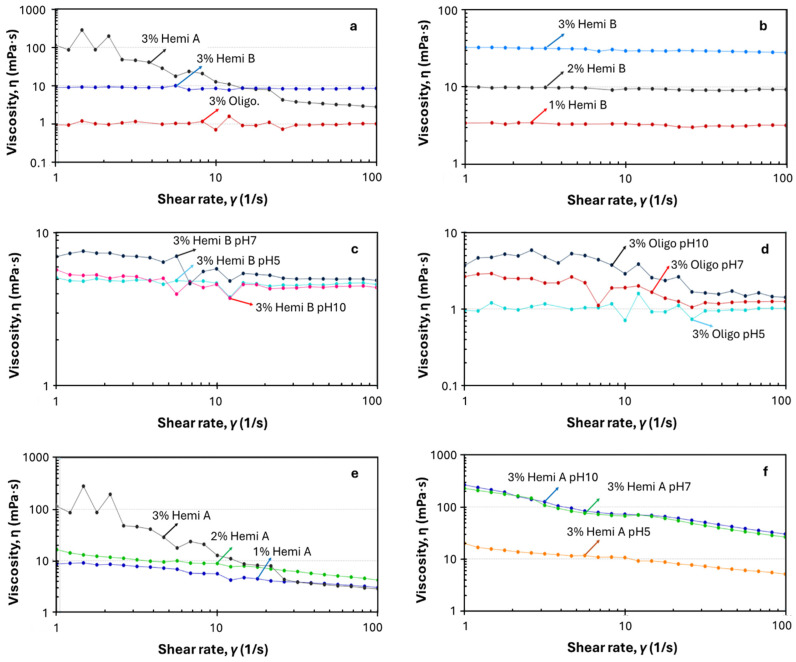
Steady shear flow curve of BSG fractions (Hemi. A, Hemi. B, and Oligosaccharides-after dialysis) at concentrations 1%, 2%, 3% *w*/*v* and pH 5, 7, and 10: (**a**) Effect of shear rate on the viscosity of 3% Hemi A, Hemi. B and Oligosaccharides; (**b**) Effect of concentration on the viscosity of Hemi B; (**c**) Effect of pH on the viscosity of 3% Hemi B; (**d**) Effect of pH on 3% Oligo; (**e**) Effect of concentration on the viscosity of Hemi A; (**f**) Effect of pH on 3% Hemi A.

Understanding the rheological behaviors of the BSG fractions is critical for guiding their functional applications. Therefore, the steady-state shear properties of aqueous solutions of Hemi. A, Hemi. B, and Oligosaccharides were examined over a range of shear rates (1–100 s^−1^). Figure 5 illustrates the change in viscosity of Hemi. A, Hemi. B, and oligosaccharides with the change in shear rate at different concentrations (1%, 2%, and 3% (*w*/*v*) and pH (4, 7, and 10) at a constant temperature. Among the Hemi. A, Hemi. B, and Oligosaccharides at (3%), Hemi. A shows the highest and the oligosaccharides the lowest, viscosity, showing the viscosity dependency on the molecular weight (Figure 5a). The molecular weight of Hemi. B isolated from cereal grains is a lot higher, 324 to 437 kDa [29], than the molecular weight of oligosaccharides generated from them. Thus, it is clear from this study that a high molecular weight Hemi B makes a more viscous solution than its lower molecular weight oligosaccharides. It is observed that the viscosity of all Hemi B (arabinoxylan) solutions increases with the increase in concentration, as shown in Figure 5b, but stays unchanged with the increase in shear rate from 1 s^−1^ to 100 s^−1^, showing a Newtonian flow behavior [30], such as most of the plant arabinoxylans [7,36]. This finding indicates that the interaction and tangling among the arabinoxylan molecules were not destroyed during the shearing, leaving their flow character unchanged.

The effects of pH are evident across each concentration of Hemi. B. At a low shear rate, the solution of 3% Hemi B at pH 7 showed a slightly higher viscosity than at pH 5 and 10. But after increasing the shear rate, 3% solution at pH 7 showed slightly lower viscosity without any change on further shearing, showing Newtonian flow behavior, such as its solutions at pH 5 and 10. Such rheological behavior at pH seven might be due to their molecular entanglement at high concentration around neutral pH, which breaks down on high shearing, giving a lower and constant viscosity. But at pH 5 and 10, this 3% solution does not show any shear thinning behavior due to no entanglement in the acidic and basic solutions. Similarly, at concentrations 1 and 2%, Hemi. B does not show any shear thinning behavior at all three pH levels, indicating that they do not have any strong molecular interaction and tangling at low concentration to be disrupted and re-aligned by high-speed shearing (Figure S3c). The 3% solution of oligosaccharide at its pH ~5 (original solution) shows a Newtonian flow behavior (Figure 5f). But the viscosity of its 3% solution at pH 7 and 10 decreases with an increase in shear rate from 10 to 100 s^−1^, showing a pseudoplastic flow behavior. In this case also, it can be interpreted that oligosaccharides entangle and associate strongly with each other at neutral and basic pH, which is disrupted at high shear rate, decreasing their viscosity (Figure 5f).

Hemi. A shows a big increase in viscosity with an increase in its concentration to 3% at low shear rate, but at high shear rate, the viscosity of its 3% solution is close to that of its 1% and 2% solutions. The viscosity of its 2% solution is slightly higher than that of its 1% solution, and at both concentrations, it shows a shear-thinning behavior (Figure 5d). This can be due to a high entanglement of Hemi. A molecule at high concentration that is disrupted by high-speed shearing. It is obvious from (Figure 5d) that at lower concentrations (1 and 2%), Hemi A exhibits slightly shear-thinning behavior, but its 3% solution shows pseudoplastic behavior. Such non-Newtonian (pseudoplastic) behavior of Hemi A at 3% concentration could be due to its structural network disruptor and reorganization, caused by external shearing forces [37]. This observation suggests that there are differences in the side-chain structures of Hemi. A and Hemi. B [36]. There is a remarkable effect of pH on 1 and 2% Hemi A solutions with the change in the shear rate (Figure S3a,b). The viscosity of 2 and 1% Hemi. A solution is higher at pH 10 and 7 than the solutions of the same concentration of Hemi. A at pH 5. Such rheological behavior of Hemi A solution might be due to its more entangled structure at neutral and basic pH than at acidic pH. At both 2 and 1% concentrations and at all three pH levels, Hemi. A shows a shear-thinning behavior with the change in shear rate from 1 s^−1^ to 100 s^−1^. Such pH-dependent viscosity and flow behavior of Hemi. A demonstration that it has highly charged molecular structures, which make them associate or repel on a change in pH.

It is quite clear from this study that there is a remarkable effect of change in concentration and pH on the rheological properties of Hemi. A, Hemi. B, and oligosaccharides. When the shear rate increased, the entangled chains were released, and physical interactions were disrupted, resulting in a decrease in their viscosity. It is believed that a more compact structure of fractions with fewer interactions could cause lower viscosity. This happens due to less resistance to flow, as there are few points at which molecules can be trapped [38].

### 3.4. Measurement of Zeta Potential

Each fraction of BSG with the appropriate concentrations was prepared as mentioned in the experimental Section 2.4. Before analysis, the solution of appropriate concentration was shaken at 200 rpm for two hours. A zeta potential analyzer was used to measure the suspension particles. Zeta potential is an important parameter that quantifies the charge developed on the particle’s surface [14]. It is all due to the ionization of chemical groups or the adsorption of ions. Zeta potential (ZP) is a physical property that is exhibited by any particle in suspension, macromolecule, or material surface. Zeta potential values are crucial for understanding the surface charge characteristics and stability of colloidal systems, nanoparticles, and suspensions. In general, the low value of zeta potential shows flocculation and aggregation. On the other hand, the high values of positive or negative zeta potential will tend to repel each other, indicating no affinity to flocculate or aggregate [14,39]. The mobility and conductivity show the variability in each fraction. Electrophoresis or electrophoretic migration analysis characterizes dispersed particles in a fluid medium under an electric field caused by a charged interface between the particle surface and the fluid. Electrophoretic mobility and zeta potential are closely related in the setting of charged particles traveling through a fluid. Electrophoretic mobility is a particle’s velocity per unit electric field applied, describing how rapidly it moves [11,39,40]. Zeta potential, electrophoretic mobility, and electrical conductivity are distinct yet interrelated concepts in electrochemistry, each defined by its own unit of measurement. Electrophoretic mobility describes how quickly a charged particle moves through a medium when exposed to an electric field. Zeta potential shows colloidal systems’ electrostatic stability, while electrophoretic mobility quantifies it in motion. The influence of concentration and pH on the BSG fractions studied is easily observed by zeta potential (Table 2). The pH is the most important factor in aqueous systems. It directly influences the ionization of surface functional groups, thereby altering the net surface charge [40]. Higher zeta potentials create a denser ionic cloud around the particle, enhancing local ion concentration and conductivity. Since a greater surface charge increases particle movement, zeta potential affects electrophoretic mobility. Particles travel with part of their ionic environment, influencing conductivity and current. Conductivity does not directly measure zeta potential or mobility, but it can indicate particle charge and electrokinetic activity [40].

On the other hand, particle interactions become more pronounced at higher solid concentrations, potentially leading to aggregation or changes in surface charge distribution. As shown in Table 2, all Hemi. A, Hemi. B and oligosaccharide fractions at different concentrations and pH show a negative net charge, which can be beneficial to use them as an additive in beverages [41]. There is no significant change in ZP with increasing concentration of Hemi. B. The absolute value of ZP decreases with increasing concentration of Hemi. A, which signifies potential for aggregation/flocculation. This effect is quite visible in the steady shear flow curve of 3% Hemi A (Figure 5e). The pH effect is so prominent when the concentration is increased in the case of Hemi A (neutral, pH 10). The zeta potential of oligosaccharides (without pH adjustment) exhibited the highest negative values as compared to Hemi. A and Hemi. B. However, after pH adjustment, the negative ZP value increased significantly in the case of both Hemi. A (pH 7 and 10) and Hemi. B (pH5, 7, and 10). This observation shows that oligosaccharides, as well as Hemi. A and Hemi. B can be used as a stabilizer in drinks and as an emulsifying agent in food products [11,14].

## 4. Conclusions

The examination of the thermal characteristics and rheological properties of BSG and its fractions will facilitate their application in various food products. Thermogravimetric analysis (TGA) revealed that Hemi A possesses superior thermal stability compared to Hemi B in inert environment circumstances, signifying its increased resistance to decomposition at high temperatures. Preliminary thermal decomposition analyses of oligosaccharides reveal a comparatively low molecular weight profile relative to other components. This conclusion arises from the premature occurrence of volatilization and degradation, aligned with the breakage of shorter-chain saccharides and glycosidic bonds. The viscosity of Hemi. A, Hemi. B, and oligosaccharide fractions of BSG were analyzed in relation to concentration, pH, and shear rate. Hemi. B demonstrated concentration-dependent Newtonian flow characteristics within the examined shear rate range. No alteration in viscosity was observed at three distinct pH levels with the increase in shear rate. Oligosaccharides produce a low-viscosity solution relative to Hemi. A and Hemi. B, exhibiting Newtonian flow characteristics in their unmodified acidic solution. However, when their solutions were neutralized to pH seven or elevated to pH 10, they exhibited pseudoplastic flow behavior. Hemi. A exhibited a flow behavior that was pseudoplastic or mildly pseudoplastic at the examined concentrations and pHs, in contrast to Hemi. B. The rheological qualities exhibited by Hemi. A, Hemi. B and Oligosaccharides suggest their potential as thickeners, gel formers, or value-added food emulsifiers. The elevated zeta potential of solutions from each fraction indicates considerable potential applications in beverage stability. This feature is also advantageous in items such as sauces and dressings. SEM-EDX examination demonstrated the presence of microporous structures consisting of C, O, N, P, Ca, and Mg on the surface of each fraction. These structures, enriched with essential elements, exhibit unique chemical functionalities and surface properties that render them advantageous additives in both biochemical processes (enhancing catalytic activity for enzyme immobilization, thereby improving reaction rates and selectivity) and thermochemical synthesis (N-doped carbon-rich materials). The fractions of BSG examined in this study exhibit their potential for several industrial applications, including utilization in biorefineries.

## Figures and Tables

**Table 1:** Surface elemental composition (wt.%) of BSG and its fractions.

Extracted Fractions	C	O	Si	Mg	Ca	P	N
BSG	65.7 ± 0.2	31.5 ± 0.2	2.5 ± 0.1	0.2 ± 0.1	0.2 ± 0.1		
Hexane extracted	62.5 ± 0.1	26.1 ± 0.3		1.0 ± 0.2	1.4 ± 0.2	3.1 ± 0.4	6.4 ± 0.3
Hemi. A	68.2 ± 0.3	17.7 ± 0.2					10.4 ± 0.2
Hemi. B	60.7 ± 0.2	28.7 ± 0.4		0.7 ± 0.3	1.5 ± 0.2	4.2 ± 0.2	
CRF	66.8 ± 0.3	29.0 ± 0.4		0.4 ± 0.2	0.9 ± 0.2	1.2 ± 0.1	
Oligo-1	63.5 ± 0.7	18.2 ± 0.2	0.6 ± 0.2	0.5 ± 0.1	1.1 ± 0.1		15.2 ± 0.3
Oligo-2	60.2 ± 0.2	20.8 ± 0.4	0.6 ± 0.2	0.7 ± 0.2	1.8 ± 0.2		15.3 ± 0.2

**Table 2:** Zeta potential of Hemi A, Hemi B, and Oligosaccharides (after dialysis) at different concentrations and pH.

Sample Name	T	ZP	Mobility	Conductivity
	°C	mV	µmcm/Vs	mS/cm
1% Hemi B	24.9	−7.33	−0.5749	0.384
2% Hemi B	25.1	−8.09	−0.634	1.29
3% Hemi B	25.0	−8.89	−0.6968	2.14
1% Hemi A	25.0	−7.77	−0.609	2.41
2% Hemi A	25.1	−3.84	−0.3009	1.31
3% Hemi A	25.1	−2.58	−0.2022	1.84
3% Oligo	24.9	−12.50	−0.9837	2.29
1% Hemi B, pH7	24.9	−17.70	−1.385	1.45
2% Hemi B, pH7	25.0	−5.28	−0.4138	2.48
3% Hemi B, pH7	25.0	−12.10	−0.9479	2.69
1% Hemi B, pH10	25.0	−10.60	−0.8309	2.22
2% Hemi B. pH10	24.9	−8.13	−0.637	2.5
3% Hemi B, pH10	24.9	−22.70	−1.781	5.78
1% Hemi B, pH5	24.9	−19.90	−1.558	0.896
2% Hemi B, pH5	24.9	−8.05	−0.6308	2.85
3% Hemi B, pH5	25.0	−12.10	−0.9462	2.34
1% Hemi A pH5	25.0	−4.70	−0.3682	1.28
2% Hemi A pH5	25.1	−6.09	−0.4773	1.35
3% Hemi A pH5	25.0	−5.57	−0.4368	1.28
1% Hemi A pH7	25.0	−13.00	−1.019	9.56
2% Hemi A pH7	25.1	−23.30	−1.825	3.02
3%Hemi A pH7	25.0	−25.80	−2.023	2.51
1% Hemi A pH10	24.9	−16.50	−1.294	3.62
2% Hemi A pH10	25.1	−28.00	−2.192	5.94
3%Hemi A pH10	25.0	−21.00	−1.645	4.09
3% Oligo pH7	25.0	−16.30	−1.275	2.45
3% Oligo pH10	25.0	−14.80	−1.162	2.57

## Data Availability

The original contributions presented in this study are included in the article/Supplementary Materials. Further inquiries can be directed to the corresponding author.

## Supplementary Materials

The following supporting information can be downloaded at: https://www.mdpi.com/article/10.3390/foods14244170/s1, Figure S1. High resolution (40k) SEM images of BSG and its fractions: a BSG; b Hexane extracted BSG; c Hemi A; d Hemi B; e CRF; f Oligo-1; and g: Oligo-2. Figure S2. SEM-EDX spectra of BSG and its fractions. Figure S3. Steady shear flow curve of Hemi A and Hemi B (a) 2 % Hemi A under different pH (b) 1 % Hemi A under different pH (c) 1 and 2 % Hemi B under different pHs.
