# Supplementary material for: Thermal, Rheological, and Surface Properties of Brewer’s Spent Grain and Its Oligo and Polysaccharides Fractions
Source: Foods. 2025 Dec 5;14(24):4170. doi: 10.3390/foods14244170 (PMC12732235; doi:10.3390/foods14244170)
Supplement: Supplementary file 1 [file foods-14-04170-s001.zip › foods-3921683-supplementary.pdf]

Thermal, rheological, and surface properties of Brewer's Spent Grain and its oligo and polysaccharides fractions

Kalidas Mainali[1], Majher I. Sarker[1], Brajendra K. Sharma,[1] Candice Ellison[1], Helen Ngo[1], Stefanie Simon[1], Madhav P Yadav,[1]*

Biobased and Other Animal Co-Products Research Unit, US Department of Agriculture, Agricultural Research Service, Eastern Regional Research Center, 600 E. Mermaid Lane, Wyndmoor, PA 19038, USA

(Paper to be submitted to foods)

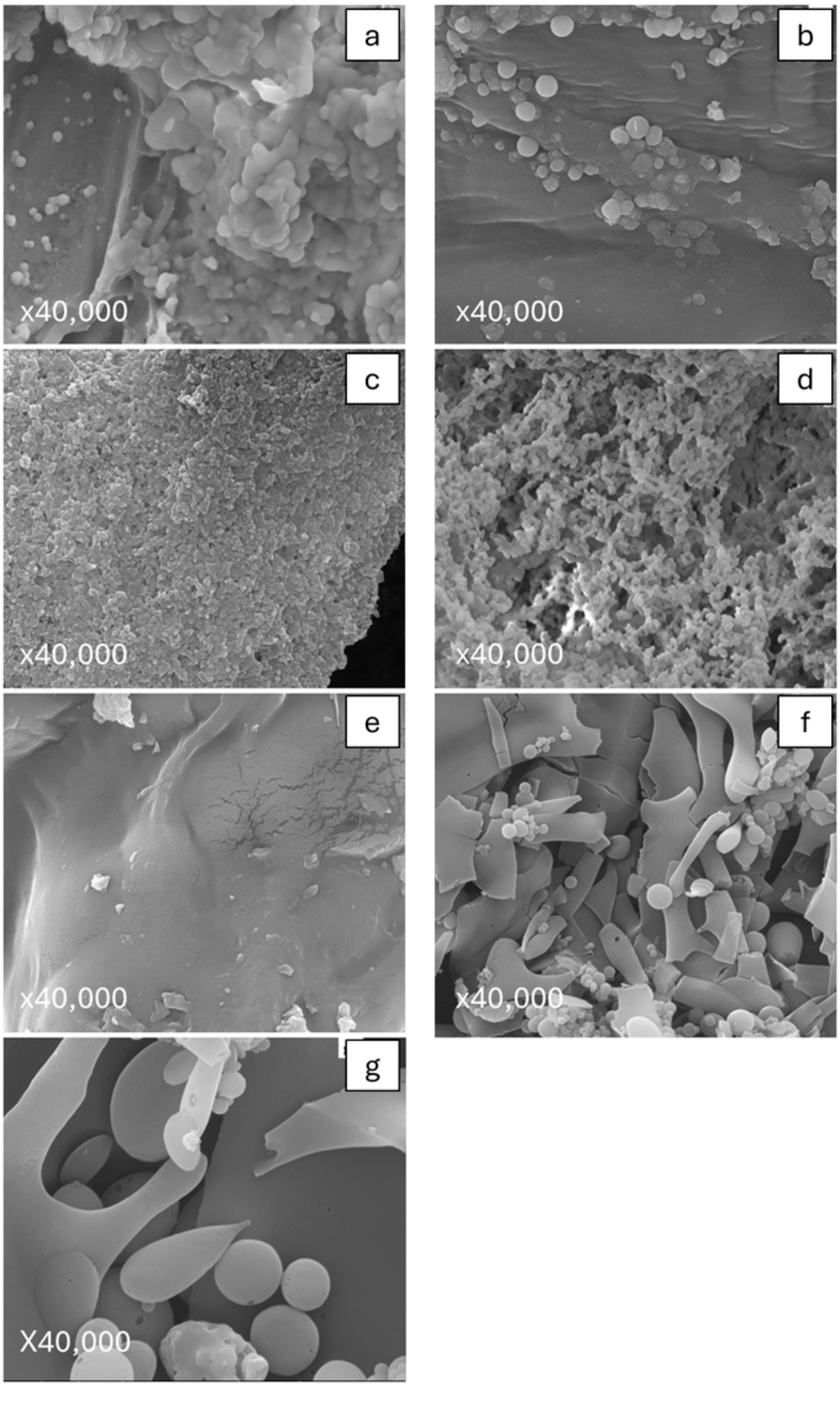

Figs S1 High resolution(40k) SEM images of BSG and its fractions: a BSG; b Hexane extracted BSG; c Hemi A; d Hemi B; e CRF; f Oligo-1; and g: Oligo-2)

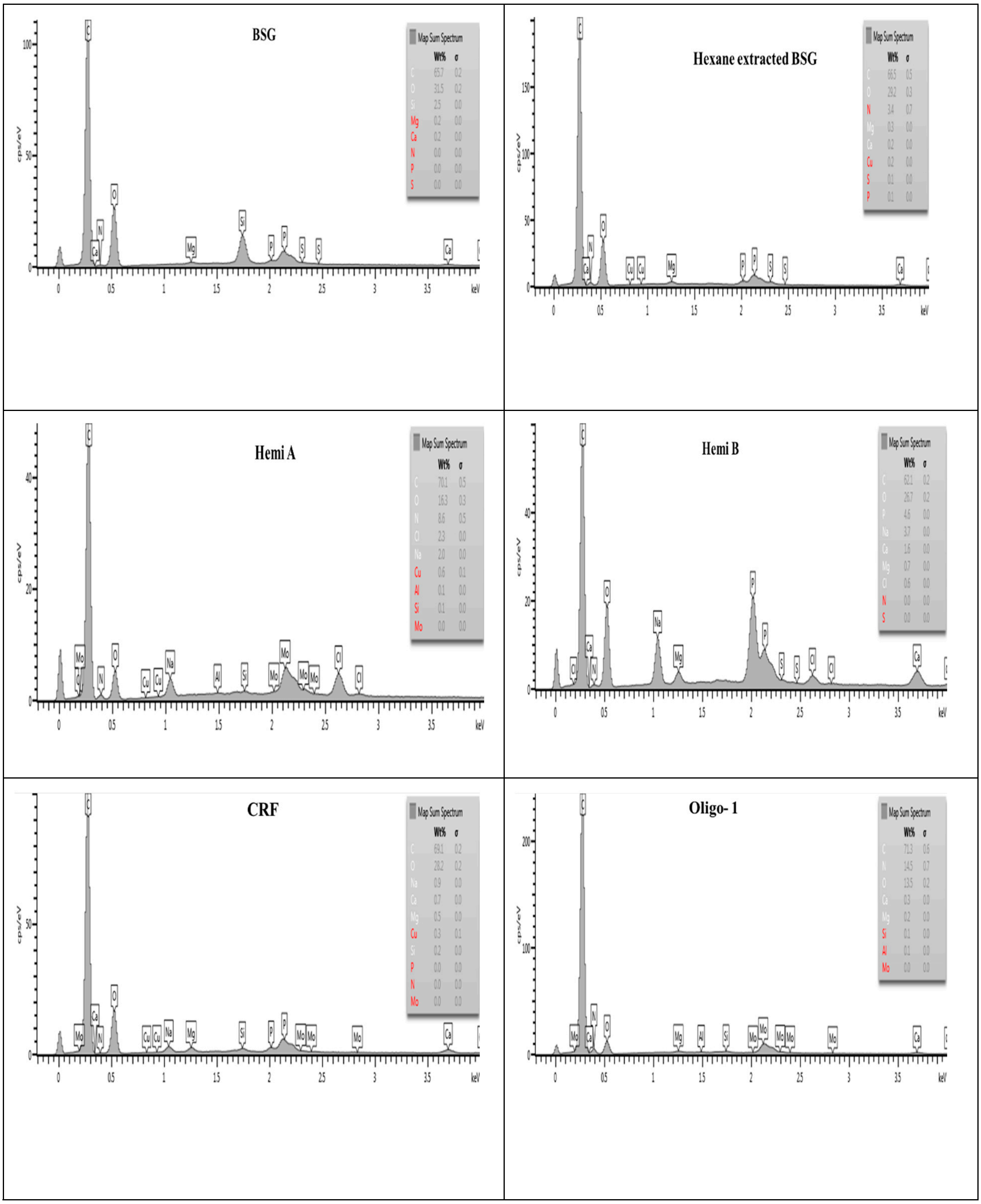
BSG
Hexane extracted BSG
Hemi A
Hemi B
CRF
Oligo- 1
Map Sum Spectrum
Wt%
σ
cps/eV
keV

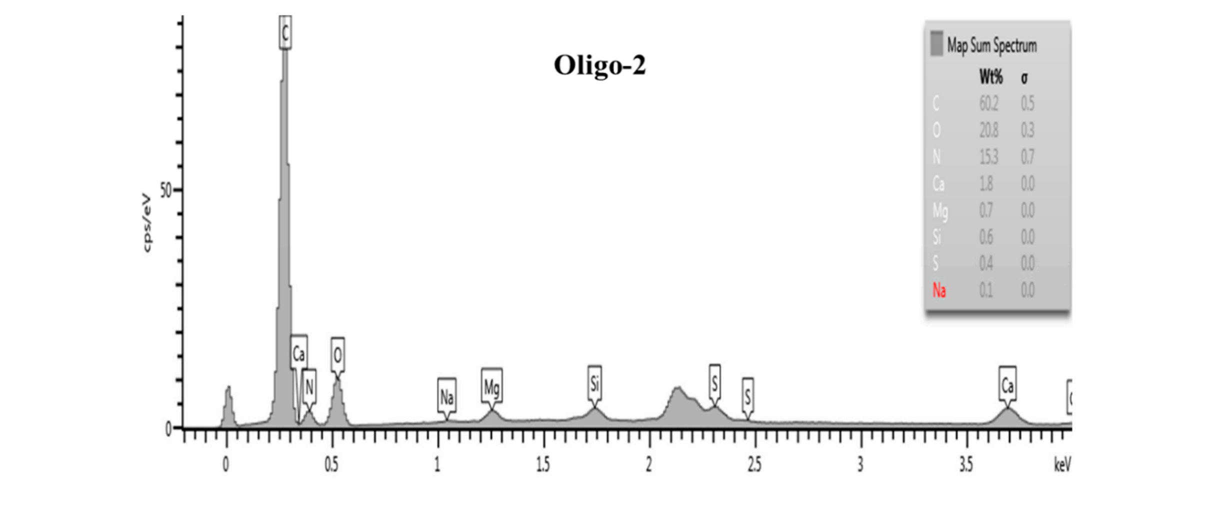

Fig. S2 SEM-EDX spectra of BSG and its fractions

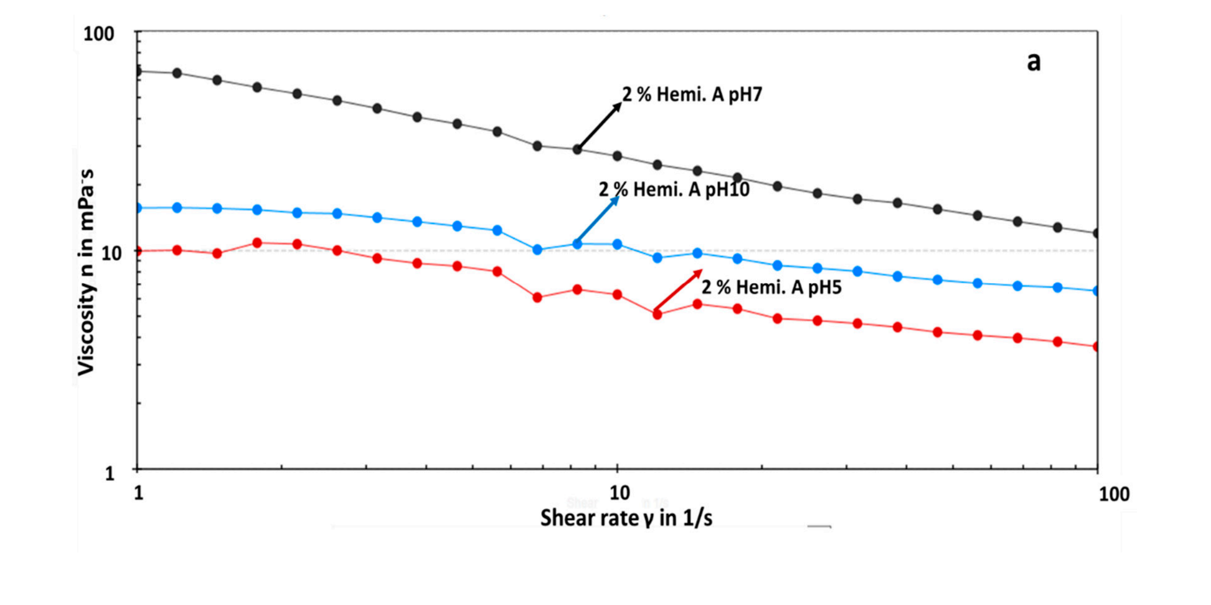

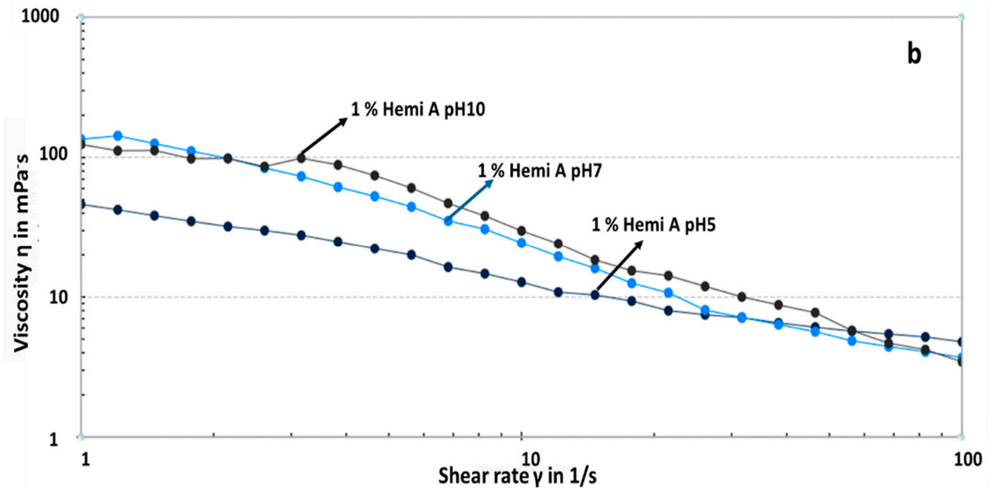

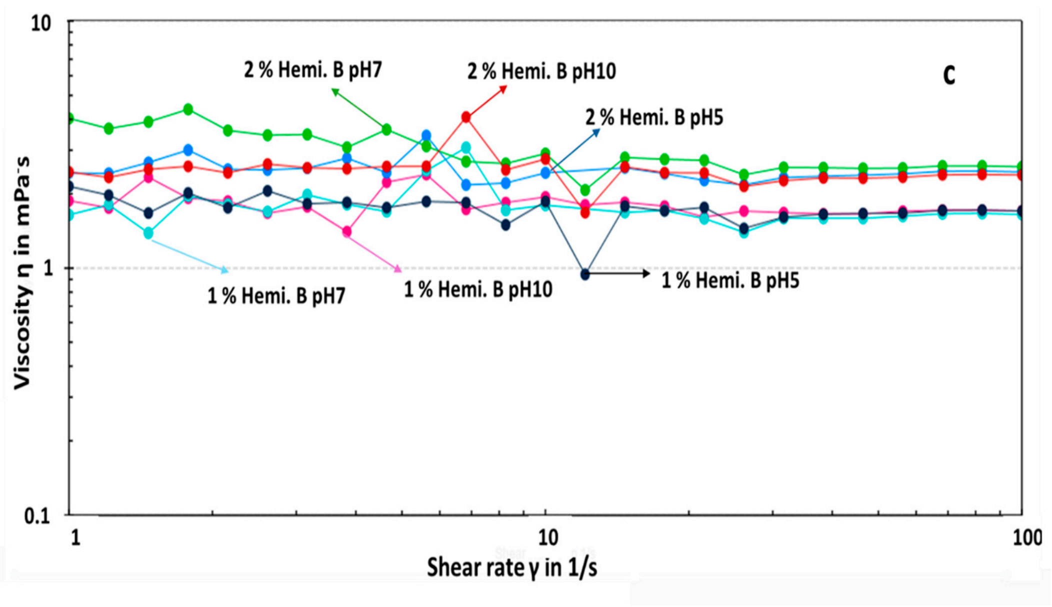

Fig. S3 Steady shear flow curve of Hemi A and Hemi B a) 2 % Hemi A under different pH b) 1 % Hemi A under different pH c) 1 and 2 % Hemi B under different pHs
